# The cancer-risk variant frequency among Polish population reported by the first national whole-genome sequencing study

**DOI:** 10.3389/fonc.2023.1045817

**Published:** 2023-02-10

**Authors:** Magdalena Mroczek, Jakub Liu, Mateusz Sypniewski, Tadeusz Pieńkowski, Bartosz Itrych, Joanna Stojak, Bartosz Pronobis-Szczylik, Maria Stępień, Elżbieta Kaja, Maciej Dąbrowski, Tomasz Suchocki, Marzena Wojtaszewska, Paweł Zawadzki, Anna Mach, Paweł Sztromwasser, Zbigniew J. Król, Joanna Szyda, Paula Dobosz

**Affiliations:** ^1^ Central Clinical Hospital of Ministry of the Interior and Administration in Warsaw, Warsaw, Poland; ^2^ Biostatistics Group, Wrocław University of Environmental and Life Sciences, Wrocław, Poland; ^3^ Postgraduate Medical Education Center, Warsaw, Poland; ^4^ Department of Experimental Embryology, Institute of Genetics and Animal Biotechnology, Polish Academy of Science, Jastrzębiec, Poland; ^5^ Department of Sports Medicine, Doctoral School, Medical University of Lublin, Lublin, Poland; ^6^ Department of Medical Chemistry and Laboratory Medicine, Poznan University of Medical Sciences, Poznan, Poland; ^7^ MNM Bioscience Inc., Cambridge, MA, United States; ^8^ National Research Institute of Animal Production, Balice, Poland; ^9^ Department of Haematology, Institute of Medical Sciences, College of Medical Sciences, University of Rzeszów, Rzeszów, Poland; ^10^ Department of Haematology, Frederic Chopin Provincial Specialist Hospital, Rzeszów, Poland; ^11^ Department of Psychiatry, Medical University of Warsaw, Warsaw, Poland

**Keywords:** genetics, cancer risk, Poland, population cancer screening, cancer

## Abstract

**Introduction:**

Population-based cancer screening has raised many controversies in recent years, not only regarding the costs but also regarding the ethical nature and issues related to variant interpretation. Nowadays, genetic cancer screening standards are different in every country and usually encompass only individuals with a personal or family history of relevant cancer.

**Methods:**

Here we performed a broad genetic screening for cancer-related rare germline variants on population data from the Thousand Polish Genomes database based on 1076 Polish unrelated individuals that underwent whole genome sequencing (WGS).

**Results:**

We identified 19 551 rare variants in 806 genes related to oncological diseases, among them 89% have been located in non-coding regions. The combined BRCA1/BRCA2 pathogenic/likely pathogenic according to ClinVar allele frequency in the unselected population of 1076 Poles was 0.42%, corresponding to nine carriers.

**Discussion:**

Altogether, on the population level, we found especially problematic the assessment of the pathogenicity of variants and the relation of ACMG guidelines to the population frequency. Some of the variants may be overinterpreted as disease-causing due to their rarity or lack of annotation in the databases. On the other hand, some relevant variants may have been overseen given that there is little pooled population whole genome data on oncology. Before population WGS screening will become a standard, further studies are needed to assess the frequency of the variants suspected to be pathogenic on the population level and with reporting of likely benign variants.

## Introduction

It has been over 20 years since the very first version of the entire human genome was released. Not a long time afterward, the scientific community agreed that cancer is a disease of the genome. Today, although advanced sequencing methods are available at a reasonable price and the role of significant genetic variants localized along the whole genome is quite well defined, the clinical implementation of whole-genome sequencing (WGS) in diagnosis and treatment remains in its infancy. Tumor sequencing (somatic variants) can provide valuable information regarding treatment response and outcomes, however, cannot explain all cancer cases. Around 5-14% of all cancers are caused by germline (inherited) variants (1). Usually, screening for germline variants related to oncological diseases encompasses only individuals with a personal or family history of relevant cancer. Here, we present the first whole-genome sequencing-based cancer population screening in the 1076 individuals from the Polish population.

Cancer remains one of the major epidemiological challenges in Poland as the number of cancer deaths is increasing. In the last 50 years, it has risen by 2.5 times, and the survival rate is one of the lowest in Europe (2). In Poland, women are affected most often by breast (22.9%), lung (9.9%), corpus uteri (7.0%), colon (5.9%), and ovary (4.3%) cancers (2). Among Polish men, the most common are prostate (20.6%), lung (16.1%), colon (6.8%), and urinary bladder (6.4%) cancers (2). The Polish population has an especially low cancer survival rate with over 10 percentage points lower than the 5-year survival rate of other European countries (3). The most significant reasons for this are a too-late diagnosis caused by the lack of screening programs and poor access to the healthcare system (4). Other characteristics of the Polish cancer-related genetic landscape are relative homogeneity and a strong influence of the founder effects (5).

The benefits of screening and preventive treatments for individuals with pathogenic cancer-related variants in different populations depend on the prevalence and penetrance of the mutation, the mortality associated with the disease, the age of the person screened, the potential effects of preventive measures on the risk of developing the disease, quality of life, and costs (6).

Some rare genetic variants have already been included in the populational screening, especially in isolated populations. Breast cancer prevention is one of the most broadly applied examples of population screening. The current screening strategy encompasses individuals with a personal or family history of relevant cancer and the criteria and screening for founder mutations in isolated populations, such as Ashkenazi Jews. In the Ashkenazi population, 3% of women carry one of the three ancient mutations: *BRCA1* 185delAG (c.68_69delAG), *BRCA1* 5382insC (c.5266dupC), or *BRCA2* 6174delT (c.5946delT) (7, 8). In Poland breast cancer mutations *BRCA1* and *BRCA2* show a strong founder effect. The incidence of the founder breast cancer mutations in the unselected early-onset breast Polish population is around 6% (8). Around 80%-90% of the *BRCA1* mutations in Polish cancer families can be explained by one of three founder mutations (5382insC, C61G and 4153delA) (6). Although the identification of carriers of hereditary breast and ovarian cancer gene variants through family cancer history alone is suboptimal, population screening is still controversial. In one of the few population screening studies, it was shown that as many as 38 of 5908 women (0.64%) carried a clinically actionable pathogenic variant (9). 42% of pathogenic variant carriers did not have a first-degree relative with breast or ovarian cancer and the family history was very often not informative (9) Polish population has been examined mainly in the context of selected genes playing a role as a control for cancer patient studies. The population prevalence for combined founder *BRCA1* 5382insC and C61G variants in Poland was 0.25% (1/400) (10). The following prevalence for *BRCA1* variants has been found: 0.17% for c.5266dupC, 0.1% for c.3819del5, and 0.08% for C61G variant. The testing strategy is suboptimal and detects only a fraction of *BRCA* pathogenic/likely pathogenic (P/LP) variants carriers (11). In the Greater London Area, only 2.6% of total estimated carriers and 5.1% of detectable carriers have been identified in the general population with current NHS strategies (12). Given the rising number of risk management options and the availability of new sequencing technologies, a future screening strategy requires an urgent discussion.

The aim of this study is to investigate germline cancer-related gene variants on the population level in Poland, to assess the type of variation (i.e., point or structural), to compare the frequencies with the frequency of corresponding variants estimated for the non-Finnish European population (NFE), and to interpret their relevance in the context of the family history-based testing. Our study is an extension of the study presented in Kaja et al. (13), however here we focus exclusively on the variants related to oncology, the frequency of these germline variants in a healthy genetically homogenous population and their potential significance on the clinical level. Further, we discuss the application of local genomic databases in oncology and the feasibility of populational cancer testing with WGS.

## Materials and methods

### The cohort

The cohort was described in detail in Kaja et al., 2022 (13). Briefly, the studied population was recruited through the project “Search for genomic markers predicting the severity of the response to COVID- 19” to the genetic predisposition to COVID-19 severity. Samples were collected from 1222 individuals of Polish origin (extended cohort), between April 2020 and April 2021. Among them 1076 were unrelated and did not suffer from any significant disease (core cohort), for a detailed cohort description see Kaja et al., 2022 (13). Samples encompassed the whole territory of Poland. All participants, guardians, or parents of the participants under 18 years of age, provided informed consent before the collection of blood samples and filling in the clinical data form, which included a questionnaire about the country of origin and chronic diseases. All participants signed informed consent. The ethical approval for the study was obtained from the Ethics Committee of the Central Clinical Hospital of the Ministry of Interior and Administration in Warsaw (decision nr: 41/2020 from 3 April 2020 and 125/2020 from 1 July 2020). The database containing anonymized genetic variants is available and can be downloaded as a VCF file from https://naszegenomy.pl/ after accepting the policy and filling in a short form.

The core cohort consisted of 1076 unrelated individuals of Polish origin. The median age of participants was 45.4 (2–99) years and there was a slight predominance of males (697 vs. 525). This is comparable to the median age in Poland. The core cohort characteristics are presented in **Table 1**. The patients have initially been recruited for the COVID studies, independent of their chronic disease status. The analysis of clinical data demonstrated that the most common chronic self-reported diseases in the survey were: hypertension (13.0%), cancer (4.6%), diabetes (4.0%), and hypothyroidism or Hashimoto’s disease (3.0%). For hypertension (13 vs. 60.28% (14)) and diabetes (4.0% vs. 6.97% (15)) the self-reported prevalence was lower than those reported in the studies, whereas for cancer mostly incidence data are available. The types of tumours identified in individuals from the extended cohort of 1222 people are reported in **Supplementary Table 1**. Because access to the phenotypic information is restricted, we were not able to extract the cancer type and genetic variant information for the core cohort of 1076 only, due to technical reasons. The information on whether cancer has been confirmed pathologically was not collected. Also, the patient reported both benign and malignant tumours under the name “cancer”. No chronic disorders were reported by 86% of participants from the core cohort.

**Table 1:** Sex and age characteristics of analyzed core cohort.

Sex	N (%)	Age ± SD
**Female**	457 (42.47%)	50 ± 17.42
**Male**	619 (57.53%)	47 ± 17.25

### Whole-genome sequencing and data processing

DNA was isolated from the patient’s peripheral blood. The library has been prepared using a TruSeq DNA PCR-free kit (Illumina Inc., San Diego, CA, USA) and 550 bp inserts. Sequencing was performed on the Illumina NovaSeq 6000 platform with the following parameters: 150 bp paired-end reads, an average read depth of 35.26×. The quality has been assessed with FastQC v0.11.7 and mapped to the GRCh38 human reference genome. Single-nucleotide variants and short indels have been identified with DeepVariant v0.8.0 and genotyped all together with GL nexus v1.2.6-0-g4d057dc. Multiallelic variant calls were decomposed into monoallelic and normalized groups using BCFtools v1.9 (16). The variants have been annotated using the following resources: Ensembl Variant Effect Predictor v.107], including references to databases of genomic variants from ClinVar v. 20220624 and dbSNP build 151, variant population frequencies from the 1000 Genomes Project, and GnomAD v2.0.1 and v3.0, as well as pathogenicity scores, such as Polyphen-2, SIFT, DANN and CADD (13). All gene coordinates were padded with variants in the 10 kb range at both ends of the genes.

### Cancer variant extraction and analysis

The variants associated with cancer were chosen based on the COSMIC database (Catalogue of Somatic Mutations in Cancer (17)) and literature review based on the PubMed search (**Supplementary Table 2**). These variants were classified as having a high, moderate, or low impact based on Sequence Ontology terms. The annotation has been performed with a SNPEff software (for details see In-depth-NGS-Data-Analysis-Course). Furthermore, the variants were filtered to identify those with known pathogenicity in cancer, following the classification available in the ClinVar (version from 2022-06-24) database. The frequency of the variants in the Polish population (this study) was compared to the frequency estimated for the non-Finnish European (NFE EUR) population obtained from the gnomAD (accessed on 2022-07-02) database. NFE population is the most similar to the Polish population. The list of variants was filtered out for moderate and high impact as a first step, and then only variants annotated in gnomAD for the NFE were further considered. For better comparison, the differences in alternative allele frequencies (AF) between the Polish cohort and gnomAD NFE were expressed in terms of odds ratio defined as $\frac{AF_{PL}}{AF_{NFE}}$ where *AF_PL_* is alternative allele frequency in polish cohort and *AF_NFE_* is alternative allele frequency in gnomAD NFE. Statistical significance of odds ratios for each variant was estimated with Fisher’s exact test and corrected for Bonferroni adjustments. The prevalence of carriers was counted as a ratio of positive samples to the total number of samples tested.

To analyze genetic diversity AF from our database have been compared to exomes from gnomAD (gnomADe), genomes from gnomAD (gnomADg) and 1000 Genomes Project (1KGP) database (https://www.internationalgenome.org/, accessed on 2023-01-13). The African (AFR), Admixed American (AMR), East Asian (EAS), South Asian (SAS) and European, gnomADe and gnomADg NFE and EUR for 1KGP, ancestries were considered. Variants were filtered according to Combined Annotation-Dependent Depletion CADD (https://cadd.gs.washington.edu/,CADD>=20), which is a deleteriousness measure.

In the manuscript the conventional variant naming has been used. The HGVS nomenclature and genomic locations are available in the **Supplementary Table 3**.

## Results

### The landscape of identified variants in the European context

**Figure 1:**
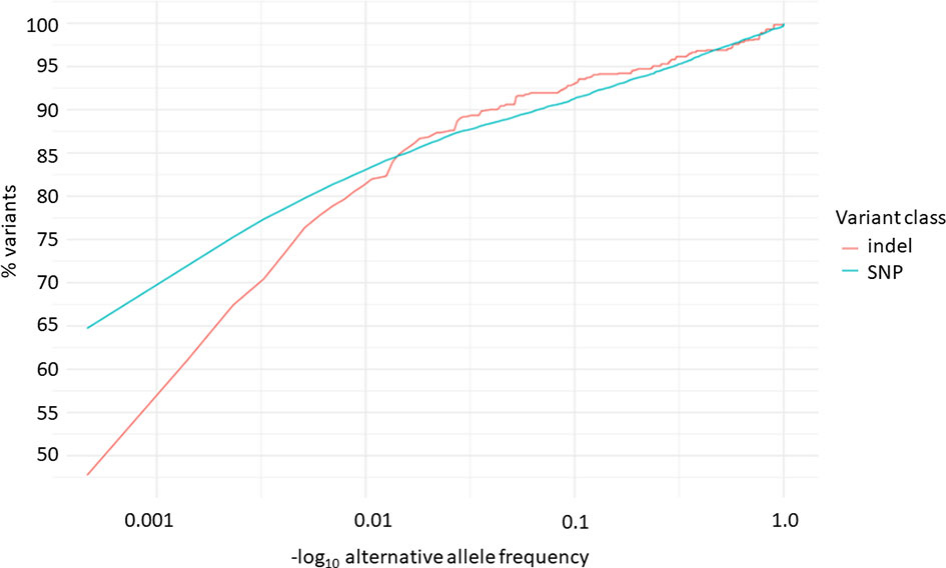
Distribution of alternative allele frequencies of indels and SNPs related to oncological diseases.

**Figure 2:**
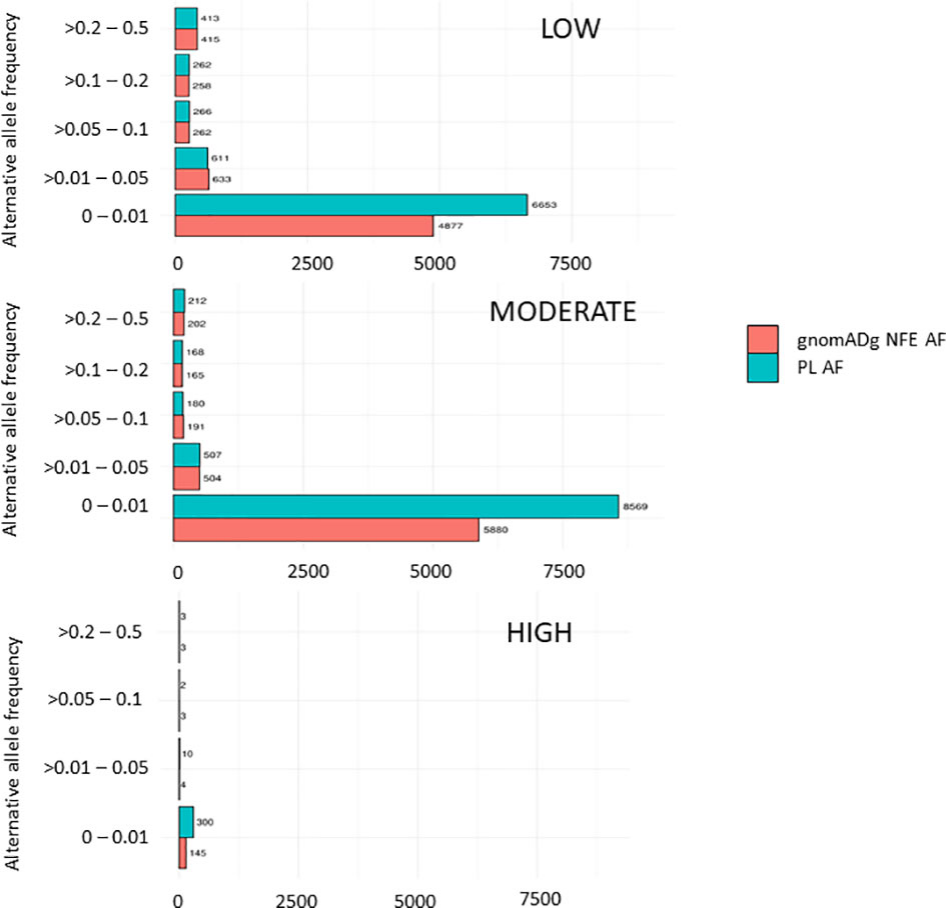
Distribution variants related to cancers in the PL population based on their impact.

Overall, 19551 variants related to oncological diseases have been identified in 806 different genes in the core cohort (**Supplementary Table 2**). Among them, 316 variants were of high impact and 9835 variants were moderate impact variants. Importantly, in our study 9739 (75.87%) of the 12836 rare variants defined by MAF< 0.01 were classified as singletons (**Figure 1**). Among the variants, 1552 were located in introns and 17353 in exons. The MAF of variants of high, moderate and low impact (as assigned by VEP) was compared between NFE (gnomAD) and Polish populations (this study). The variant enrichment divided into medium and high frequency is presented in **Figure 2**. High-impact variant for *FANCC* had a significant OR (p<0.05) in the Polish population. Moderate impact variants with the highest OR (p<0.05) in the Polish population were: *NUTM2B, MUC4, NOTCH1, NUTM2B, USP6* (**Table 2**). Their functions and pathways they are involved in are presented in **Table 3**.

**Table 2:** High and moderate impact variants with a significantly higher frequency in the PL population as compared to the gnomAD in NFE population.

High impact													
position	Gene name			CADD score		Frequency in PL (current study		Frequency gnomAD in non-Finnish population (NFE)		OR		p.value	
chr9_95126582_C_G	*FANCC*			33		0.0023		1.80E-05		130		4.50E-08	
Moderate impact													
chr10_79706060_C_T	*NUTM2B*			8.255		0.039		1.20E-04		310		4.00E-124	
chr3_195779189_C_G	*MUC4*			17.14		0.012		5.40E-05		220		4.00E-38	
chr9_136515636_C_T	*NOTCH1*			10.07		0.0019		1.00E-05		210		5.90E-07	
chr10_79706584_A_G	*NUTM2B*			2.49		0.023		1.20E-04		180		1.20E-68	
chr17_5132920_G_A	*USP6*			3.634		0.0023		1.80E-05		130		4.50E-08	

Only variants described in gnomAD in non-Finnish population are considered. Significance threshold was defined as Bonferroni α < 0.05.

**Table 3:** Protein's function, pathways and types of cancers related to the high and moderate impact variants with the highest OR.

Gene	Type of cancer associated	Protein function	Related pathways	% of cancers mutated	Other diseases associated in variants in the gene
*FANCC*	kidney cancer, skin cancer, cancers of the upper aerodigestive tract, colon adenocarcinoma, lung adenocarcinoma,	multifunctional protein involved in the suppression of cell death in response to a wide range of stimuli including DNA-crosslinking agents, factor withdrawal, dsRNA, stimulatory cytokines and Fas ligation, as well as a having a possibly interrelated role in maintaining of the redox state of the cell.	DNA damage/repair	0.97	Fanconi anemia
*NUTM2B*	stromal sarcoma	–	–	–	–
*MUC4*	breast cancer, leukaemia, pancreatic cancer	encodes an integral membrane glycoprotein found on the cell surface	–	–	–
*NOTCH1*	colon adenocarcinoma, lung adenocarcinoma, breast invasive ductal carcinoma, endometrial endometrioid adenocarcinoma, and skin squamous cell carcinoma	takes part in in multiple developmental processes and the interactions between adjacent cells	Signalling Pathways Signalling by NOTCH Pre-NOTCH Expression and Processing Pre-NOTCH Transcription and Translation Pre-NOTCH Processing in Golgi	4.48	Adams-Oliver syndrome
*NUTM2B*	kidney sarcoma soft tissue sarcoma			–	
*USP6*		Involved in protein deubiquitination and regulation of vesicle-mediated transport.	Arf6 signalling events	0.16	

Description based on the https://www.ncbi.nlm.nih.gov/gene/ and https://www.mycancergenome.org/. -, no information found.

### Polish population in the global context

To present variants related to oncology identified in our database in regard to global genetic diversity and other genetic and genomic databases, we compared the number of variants identified in the Polish population to different ancestries and from three databases. In the first step the analysis was performed for the *BRCA1/BRCA2* P/LP variants identified in the our database in the Polish population. In the context of 1KGP database, all these variants were unique across ancestries. The number of P/LP *BRCA1/BRCA2* variants shared according to gnomADg between Polish population (this study) and other ancestries presented as follows: NFE (n=5/7), AMR (n=3/7), AFR (n=2/7), EAS (n=1/7), SAS (n=1/7).

**Figure 3:**
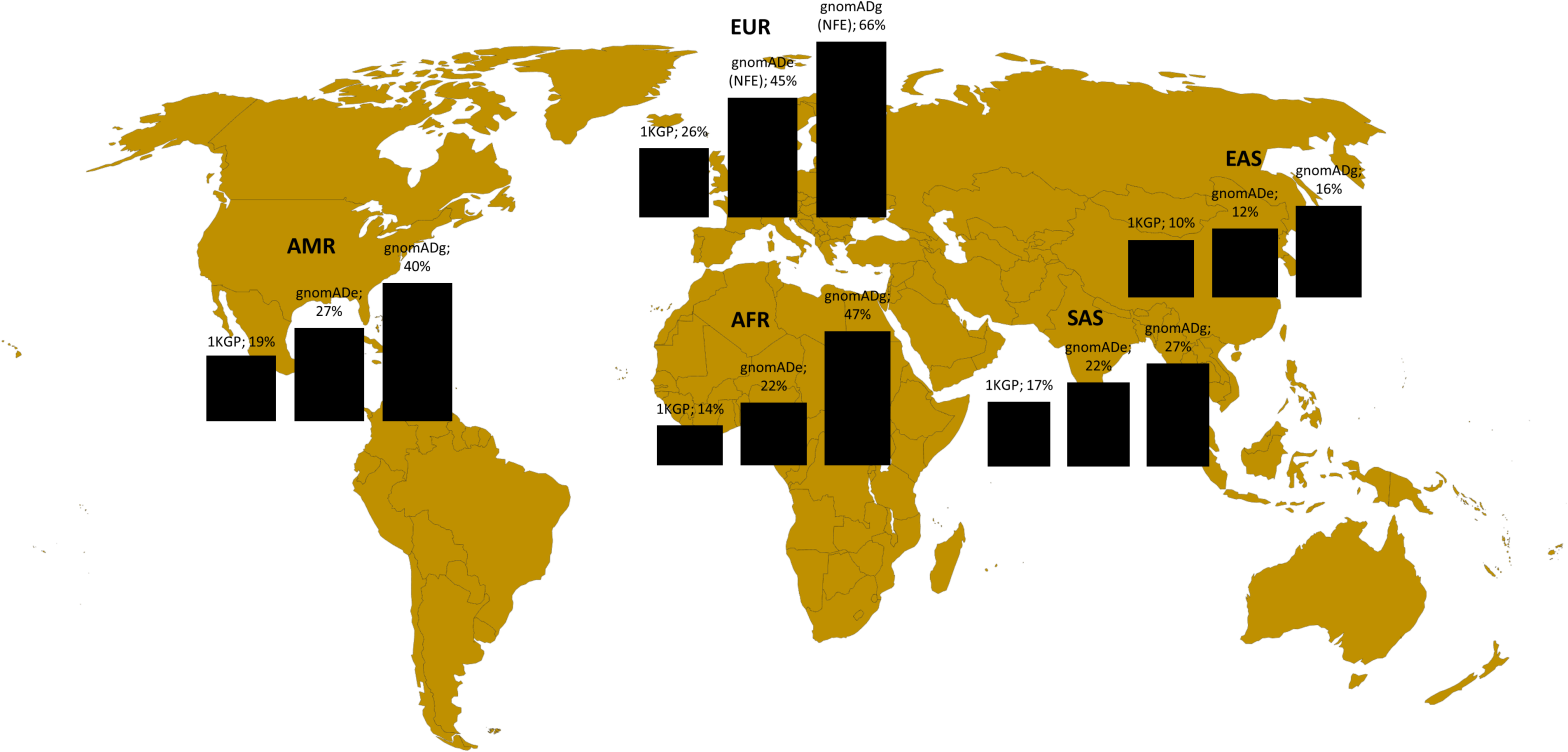
Variants shared between Polish population (this study) and different ancestries according to 1KGP, gnomADe and gnomADg. Legend: AFR- African, AMR- Admixed American, EAS-East Asian, SAS-South Asian and EUR (European-EUR for 1KGP and NFE-European non-Finnish for gnomADe and gnomADg).

In the second step, we analyzed the variants related to oncology in Poland in the broader perspective. Not to exclude common variants, but at the same time to use computational resources effectively, we analyzed the variants with CADD > = 20 from the variants related o oncological diseases list. These included 8491 variants, among them 840 rare (MAF<0.01) and 7651 common (MAF>0.01). Among 8491 variants, 5647 were unique for Polish population (monomorphic) according to 1KGP database, 4392 according to gnomADe and 2315 according to gnomADg. The number of variants related to oncology shared between Polish cohort and particular ancestries is shown in **Figure 3**.

### Variants in BRCA1/BRCA2

In the Polish population, 45 different rare variants were identified within the *BRCA1* gene and 72 in the *BRCA2*. Among them in *BRCA1* two were of a high impact, 30 of a moderate impact and 13 of a low impact. Among *BRCA2* five were of high impact, 46 of moderate impact and 21 of low impact. According to ClinVar three variants in *BRCA1* were classified as P/LP (c.5266dupC, 3819del5, and c.181T>G (p.Cys61Gly)) and four variants in *BRCA2* (c.658_659delGT, c.1813dupA, c.5238dup and c.9371A>T) were classified as P/LP. The *BRCA1/BRCA2* variants and their AF are shown in **Supplementary Table 4**. Altogether, the AF for the *BRCA1/BRCA2* P/LP variants according to ClinVar (18) in the unselected populations of 1076 patients in a genetically homogenous Polish population was 0.42%. That means that we identified 9 P/LP *BRCA1/BRCA2* variants in the unselected Polish population. AF for the three pathogenic *BRCA1* founder variants was 0.23%. None of the *BRCA1/BRCA2* variants reached a statistically relevant higher allele frequency in the Polish population comparing to other European populations, suggesting that this kind of studies require a much larger cohort.

### Variants in non-BRCA1/BRCA2

Several genes in which the variants were identified, are related to hereditary cancer syndromes, such as *PALB2*, related to ovarian and breast cancer or *JAK2* (c.3323A>G, p.Asn1108Ser) associated with lung adenocarcinoma, myeloproliferative neoplasm, breast invasive ductal carcinoma, polycythemia vera, and colon adenocarcinoma (**Supplementary Table 5**). *RAD51* protein product is involved in DNA repair and is altered in 0.65% of all cancers with lung adenocarcinoma, glioblastoma and glioblastoma multiforme (19). Germline mutations in the *CDKN2A* strongly predispose to cutaneous melanoma (16, 20). Although we identified a variant (c.194-3652G>C) in the *CDKN2A* gene located only one bp distant from the pathogenic *CDKN2A* variant c.-34G>T (21, 22), after a thorough analysis the variant proved to be benign. Other variants reported as pathogenic/likely pathogenic/with conflicting interpretations of pathogenicity according to the ClinVar were located in the following genes: *MSH6* associated with Lynch Syndrome i.e. hereditary non-polyposis colorectal cancer (23), *PIK3CB*, which is altered in 1.62% of all cancers such as colon adenocarcinoma, prostate adenocarcinoma, lung adenocarcinoma, breast invasive ductal carcinoma; *RPN1* altered in 0.19% of all cancers with high grade ovarian serous adenocarcinoma, colon adenocarcinoma, oesophageal adenocarcinoma, bladder urothelial carcinoma (24). Although the variants in these genes have been described as enriched in cancer populations or considered pathogenic by expert panels, the evidence of variant pathogenicity is often reported in very small sample sizes comprising a few families in control and a diseased group and thus not being representative of the whole population.

### Phenotype correlation

Variants in 5 genes (*RPN1, CDKN2A, HRAS, PALB2, CBFA2T3*) have been identified in 32 individuals with tumor from the extended cohort and classified as pathogenic, likely pathogenic, and VUS **(** **Supplementary Table 1**). Given the limited information on cancer in our study cohort, e.g., in 4 cases there is no information about the cancer type, it is difficult to correlate the cancer type with genetic variants. Variants in three genes (*RPN1, HRAS* and *PALB2*) were carried by several individuals with cancer in extended cohort. Ribophorin 1 (RPN1) is a major part of oligosaccharyltransferase complex, which takes part in the glycosylation process. *RPN1* is upregulated in breast cancer and has also been associated with several other cancers, such as high-grade ovarian serous adenocarcinoma, colon adenocarcinoma, oesophageal adenocarcinoma, and bladder urothelial carcinoma. As much as 7 out of 12 individuals from our cohort with cancer had a likely pathogenic variant on both alleles. Another recurrent variant was localized i*n HRAS* gene. HRAS is a known oncogene related to several different cancers, among them Costello syndrome, a rare condition predisposing to tumours in different parts of the body, epidermal tumours, head and neck cancer, bladder carcinoma, and with ovarian carcinoma patients, but was also identified in the healthy controls The variants frequency is not annotated in ClinVar so that it also requires further characterization on the populational level. The variant p.Ile1035Val in *PALB2*, associated with breast and ovarian cancers, was identified in two out of eight individuals with breast cancers and in one ovarian cancer person from the extended cohort. All these variants will be further followed up in the affected cohorts in Poland in further studies.

## Discussion

Oncology remains the major field that can benefit most from whole genome sequencing (25). The first collection of over 350 cancer-related genes has been created, protein-coding genes to be more specific, with new genes being added over time (26). More and more WGS is being performed in a variety of different populations, supporting the notion that WGS of different cohorts enhances the power to identify genetic associations (24, 27–30). Recently, in light of rising population consciousness in terms of cancer population testing and with sinking costs of genetic testing it has been suggested that genetic testing should be offered on the population level. Cancer-susceptibility-gene (CSG) testing might shift from bespoke tests towards whole genome or whole exome analysis as a part of comprehensive population-wide programmes; incorporating such strategy into healthcare systems, with equitable access for the entire population, will be challenging. These issues should especially be discussed, together with variant-of-unknown-significance (VUS) interpretation, penetrance, and genetic counseling. The Thousand Polish Genomes database can find several applications in oncology and show benefits in comparison to the other genomic databases in the following areas: analysis of the intron variants, calculating a polygenic risk score (PRS) and being a reference database for the Polish studies on cancer patients.

Traditionally, most of the studies were focused on the identification of cancer mutations solely in protein-coding genes, ignoring the remaining 99% of the genome. Gene panels can detect only mutations in 0.01 – 0.10% of the genome, at best. The main reason for it was that a large proportion of early NGS-based studies was constrained to whole exome sequencing - a more cost-effective way of ascertaining large samples, as well as the fact that the functional annotation of the non-protein coding part of the genome has constantly been updated and thus made more informative (31). In our study, 89% of the variants in cancer-related genes were identified in non-coding regions, although most of the database reported variants derived from the exons. Whole Genome Sequence (WGS) based analyses of thousands of genomes representing various cancer tissues revealed multiple cancer-driver events localised in non-coding regions of DNA such as promoters and introns, to name just a few. Such events include not only single nucleotide variations (SNV) but also small indels and larger structural changes (32). There are already some attempts to better understand the function of the non-coding regions. For example, on the large UK Biobank sample a depletion rank has been applied to characterize the function and importance of the non-coding variants. Surprisingly, among the 1% of regions with the lowest DR, 13.0% were coding and 87.0% were non-coding (33). This suggests the still not understood importance of non-coding regions.

Rare variants in high/medium penetrance cancer-related genes, although significant, cannot explain the cancer genetic risk sufficiently. Some common variants (polymorphisms, SNPs with a frequency >1%) may contribute to the individual high or moderate risks of developing cancer or alternatively may imply a lower risk of developing the malignant disease. Polygenic Risk Score (PRS) aggregates the effects of many genetic variants across the human genome into a single score, being an estimate of an individual’s genetic liability to a trait or disease (34). PRS can be useful in the prediction of the progression and recurrence of cancer, which allows improving the efficiency of population-level screening. Recent studies showed that common variants may sufficiently assess personal risk, at least for some types of cancers, such as breast, colon, lungs, thyroid and prostate (35). For prostate cancer the combination of rare and common genetic variants has been reported to be the most efficient in the disease risk prediction (36). Black et al. (37) reported that a set of 72 SNPs PRS was predictive of prostate cancer and could be used to identify unaffected individuals at high risk of developing prostate cancer. In the case of breast cancer, Kapoor et al. (38) highlighted that the lifetime risk of breast cancer associated with classical risk factors was greater for women with higher PRS. The estimation of genome wide PRS for breast and prostate cancers showed that having a high PRS led from 21% to 38% higher lifetime risk, and 4 to 9 years earlier disease onset. PRSs improved model calculations over age, sex and clinical risk and substantiated the application of PRS in population screening (39). PRS may additionally play the role of biomarker or response markers for chemotherapy and be taken into consideration to reduce the costs of cancer screening (34). There are also critical voices on the use of PRS, especially in a clinical setting. Before being introduced to the clinics, PRS must provide significant discrimination and be applicable in terms of early detection and prevention. One of the concerns regarding PRS is the fact that most analyses performed, are based on European populations and may not be generalizable to other (e.g., Asian) populations (40). That is why understanding the cancer-related variants in the context of the country’s population is crucial for the interpretation at the population level and later on in an individual context. Although the data gathered as part of this study do not include at the moment strictly cancer patients, they can, later on, serve as a population control for assessing PRS.

To check the representation of the variants in genes related to oncology in the Polish cohorts, we compared them to the data of multiple ancestries from the global databases. For the *BRCA1/BRCA2* analysis, the pathogenic variants identified in this study for the Polish population were mostly shared with the NFE ancestry and just one common variant within the EAS and SAS ancestry was identified. As Polish *BRCA1/BRCA2* P/LP variants have a strong founder effect that is why as expected there are shared mainly with the European NFE population, which is the closest genetically. Most of these variants are not represented in the other ancestries. In the context of variants with a highest CADD (mostly common variants) located in the genes associated with cancer, when compared to the gomADg database, encompassing most of the variants, as many as 27% were monomorphic for Poles, but this was strongly dependent on the database. The result highlights the importance of creating a local database due to the fact that a large part of the variants related to oncological diseases is population specific and not shared not only between the various ancestries, but also between the same continent (PL vs. NFE). Moreover, the size of the database has a considerable meaning, while a low number of individuals can lead to bias. For example, 1KGP database encompasses around 3202 WGS individuals from different ancestries (41), while gnomAD has aggregated 15.708 genomes and 125.748 exomes (42). Although 1KGP database has several advantages, such as a broad representation of the human genetic variation among ancestries and inclusion of only healthy individuals, however considering only a small number of individuals may not properly illustrate the similarities and diversity between different ancestries in terms of germline variants related to cancer.

The Thousand Polish Genomes database can be used as a reference database for germline cancer variants. To the best of our knowledge, this is the first study on the Polish population using a whole-genome sequencing approach for cancer predisposition screening. Previous studies focussed on selected genes and known founder mutation, although they have encompassed large cohorts, such as 16 229 healthy persons. The Thousand Polish Genomes database encompass also genetic modifiers and genetic biomarkers related to treatment outcomes. In Poland several projects targeted the discovery of new genes related to cancer and cancer susceptibility genes (43, 44). The Thousand Polish Genomes database can provide population references for such studies. Also, recently it has been shown that it may be possible to construct germline variants from the discarded tumor sequencing reads (45). The local population can help to validate such a result. One of the most relevant issues is in assessing variant pathogenicity. This is a challenge also for the variants already reported in ClinVar. For example, the variant in *PALB2* (c.3103A>G (p.Ile1035Val)) gene reported as having conflicting interpretations of pathogenicity in ClinVar and suggested of being pathogenic in the literature (46) was present in healthy individuals as well as in breast cancer individuals from the extended cohort Our study confirmed the presence of this *PALB2* (c.3103A>G (p.Ile1035Val)) variant in the Polish core cohort in a very high AF (12.3%).

Are we ready for population cancer screening using WGS? Given the sinking costs of sequencing technologies and the rising availability of prevention management alternatives, we are heading towards it. We identified nine P/LP variants in the *BRCA1/BRCA2* variants only in the unselected Polish population. However, especially on the individual level we are not able to interpret data reliably, for example in terms of PRS, and that is why we cannot make any prediction based on them. As a scientific and clinical community, we should further try to provide results of the negative association in databases like ClinVar, not only the positive ones. We should be aware of differences between populations, even within the same ethnicity group, and to be able to identify risk alleles we advocate for large cohorts. For now, cancer screening with WGS on a research basis in larger populations will show us how feasible the method could be in clinics.

## Data availability statement

The datasets presented in this study can be found in online repositories. The names of the repository can be found below: https://1000polishgenomes.com.

## Ethics statement

The studies involving human participants were reviewed and approved by the Ethics Committee of the Central Clinical Hospital of the Ministry of Interior and Administration in Warsaw (decision nr: 41/2020 from 3 April 2020 and 125/2020 from 1 July 2020). The patients/participants provided their written informed consent to participate in this study.

## Author contributions

Conceptualization, MM, PD, BI, JL. Formal analysis, MM, TS, JL. Data curation, TP, PD, PT, TS, JSt. Data generation, PD, PT, PZ, ZK, EK, MD, MW. Methodology, MM, MatS, TS, JSt, BP-S. Visualisation and writing—original draft preparation, MM, JL, BI, JSz, BP-S, MarS. Writing—reviewing and editing: JSz, PD, JSt, MD, BP-S, TP. Funding acquisition, ZK. Supervision: PD, JSz. All authors contributed to the article and approved the submitted version.
